# Primer and interviews: Molecular mechanisms of morphological evolution

**DOI:** 10.1002/dvdy.22454

**Published:** 2010-11-09

**Authors:** Julie C Kiefer

**Affiliations:** Department of Neurobiology and Anatomy, University of UtahSalt Lake City, Utah

**Keywords:** morphological evolution, parallel evolution, genetic regulatory network, deep homology, pleiotropy, *cis*-regulatory hypothesis, coding hypothesis

## Abstract

The beauty of the developing embryo, and the awe that it inspires, lure many scientists into the field of developmental biology. What compels cells to divide, migrate, and morph into a being with a complex body plan? Evolutionary developmental biologists hold similar fascinations, with dynamics that take place on a grander timescale. How do phenotypic traits diverge over evolutionary time? This primer illustrates how a deep understanding of the basic principles that underlie developmental biology have changed how scientists think about the evolution of body form. The primer culminates in a conversation with David Stern, PhD, and Michael Shapiro, PhD, who discuss current topics in morphological evolution, why the field should be of interest to classic developmental biologists, and what lies ahead. Developmental Dynamics 239:3497–3505, 2010. © 2010 Wiley-Liss, Inc.

## EVOLUTION OF A HYPOTHESIS

No one could mistake a human forelimb for a bird wing. Or could they? After peeling back layers of skin and muscle, one finds the same basic skeletal elements in both: scapula, humerus, radius and ulna, wrist, and digits. This morphologic homology supports the theory that the species' appendages stem from a common ancestor. However, even conserved skeletal elements vary in design. For example, to aid in flight, its wrist and digit bones are fused. Whale flippers also share the basic skeletal elements that birds and humans do, but they have additional phalanges for added length and flexibility. For decades, experimental evidence supported the then prevailing notion that the great variation observed among homologous traits is driven by mutations in the protein-coding regions of genes (Hoekstra and Coyne,^2007^; Stern and Orgogozo,^2008^).

Intuitively, protein-coding sequence would seem to be the most fertile ground from which mutations can execute changes in gene function. After all, in one fell swoop, a nonsynonymous change in amino acid sequence, or a large-scale genetic change such as gene loss, rearrangement, or duplication, can produce immediate effects. These changes can alter protein structure, stability, activity, and/or produce genes with novel function. Nevertheless, the so-called “coding mutation hypothesis” has fallen out of favor in recent years.

## (R)EVOLUTIONARY DEVELOPMENTAL PRINCIPLES

The eventual demise in popularity of the coding hypothesis can be traced in part to the molecular techniques explosion of the 1980s. The ability to rapidly and thoroughly analyze DNA sequence and gene expression, and to manipulate gene expression and function, revealed that there are several core principles that guide much of embryonic development. Described below are three of the key principles that produced ripple effects that extended beyond classical developmental biology, eventually re-shaping the theory of phenotypic evolution.

### Deep Homology: Making the Most of Pre-existing Information

**Fig. 1:**
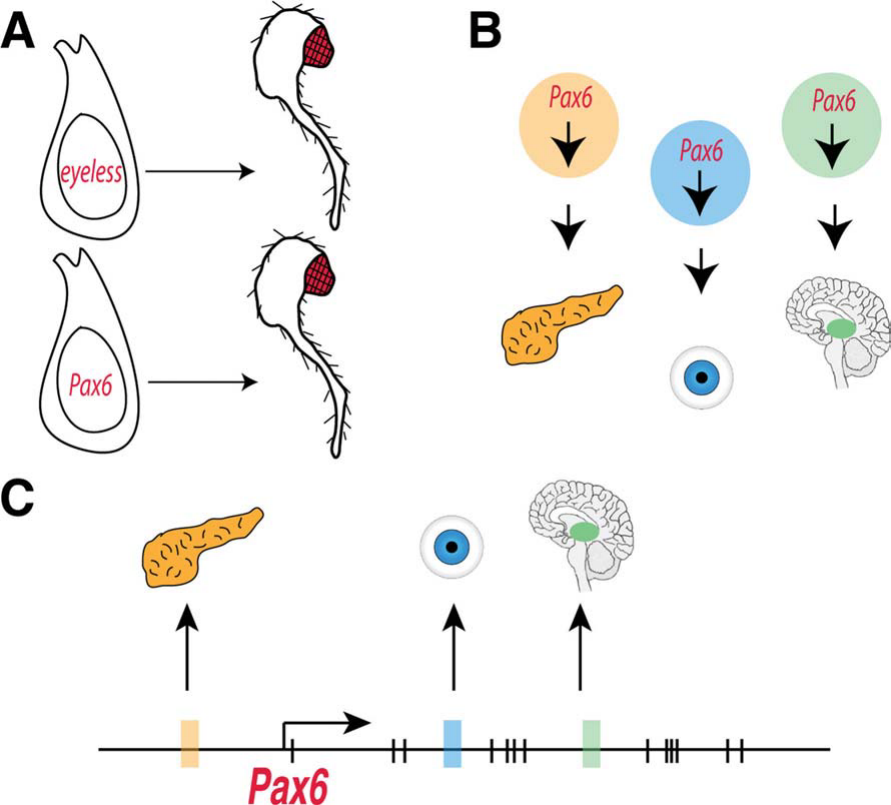
Three developmental biology principles that shaped the theory of morphological evolution. **A:** Deep homology. Expression of ectopic *Drosophila eyeless* or its ortholog mouse *Pax6* in leg imaginal discs induces formation of ectopic eye structures on legs, suggesting the function of *eyeless/pax6* is conserved in development of analogous compound and single lens eyes. **B:** Mosaic pleiotropy. Mouse *Pax6* regulates pancreatic, eye, or brain development depending on the genetic milieu in which it is expressed. **C:** Modular *cis*-regulatory enhancers (CREs). *Pax6* CREs direct expression in many sites including the pancreas, neural retina, and diencephalon, the latter two of which reside within *Pax6* introns.

The discipline of evolutionary development (evo-devo) was born from the discovery that single genes, and even entire gene networks, retain similar functions across species. One of the first examples illustrating the concept was the discovery that orthologs of the paired box transcription factor, Pax6, regulate development of two vastly different eye types: the vertebrate single lens eye and the *Drosophila* compound eye. *Drosophila eyeless* (*ey*) and mouse *Pax6* not only share extensive sequence identity and similar expression patterns, but they are to a certain extent functionally interchangeable. The latter is demonstrated by the striking finding that misexpression of either mouse or fly *Pax6* in *Drosophila* imaginal discs induces ectopic ommatidia (Fig. 1A; Halder et al.,^1995^). What's more, although there are clear differences between mouse and *Drosophila* gene networks that regulate eye development, the networks do have several genes in common such as *eyes absent/Eya* and *sine oculis/Six* (Hanson,^2001^). Based on these and similar findings, the phrase “deep homology” was coined to describe the concept that analogous structures are derived from similar genetic mechanisms (Shubin etal.,^1997^).

Beyond similar structures, regulatory genetic circuits can also appear in diverse anatomical contexts. *Pax6* null mice also fail to form lens as well as nasal placodes (Hogan etal.,^1986^). Of interest, nasal development also involves shared expression of *Pax6*, *Six*, and *Eya* (Purcell et al.,^2005^). Therefore, it is tempting to speculate that deployment of the gene program during nasal development may represent co-option of an ectodermal placode based program at a novel anatomical site. These and other data suggest that rather than building novel structures from scratch, nature instead creates morphological diversity by modulating existing information (Shubin et al.,^2009^).

### Mosaic Pleiotropy: The Cons of Recycling

Many of the key players in conserved gene regulatory networks are “toolkit genes,” transcription factors and signaling molecules that are deployed repeatedly throughout embryogenesis. Their ultimate function depends upon when and where they are expressed, whether they have been integrated into different genetic regulatory networks, and other variations in the genetic landscape (epigenome, transcriptome, etc.). For example in addition to eye development, Pax6 also regulates neural, pancreatic, and pituitary development (van Heyningen and Williamson,^2002^). As important developmental regulators, toolkit genes are poised to shape morphological evolution.

The question of how to change a gene that plays several developmental roles is a tricky one. Changes in coding sequence could wreak pleiotropic consequences that would likely be detrimental to the fitness of the organism. By extension, the common belief that gene duplication and divergence is a mechanism of change is also unlikely because developmental processes are generally sensitive to changes in gene dosage (Carroll,^2008^). On the other hand, since genes that regulate physiological processes (i.e., metabolism, enzyme function, etc.) tend to reside at the terminal end of genetic regulatory networks, an animal may better tolerate these types of mutations in physiological genes (Stern and Orgogozo,^2008^). Therefore, because the rules of the evolution of function may differ from the rules of the evolution of form, coding mutations are perhaps less likely to be driving forces of phenotypic evolution.

### Modular *cis*-Regulatory Enhancers: A Means for Incremental Change

If not by coding changes, then what are the molecular mechanisms of phenotypic evolution? The answer lies in the observation that modular *cis*-regulatory elements (CREs) govern the complex expression pattern of many toolkit genes. Again using mouse *Pax6* as an example, its expression is regulated by multiple enhancers: individual CREs direct expression in the pancreas, neural retina, diencephalon, and other sites (van Heyningen and Williamson,^2002^; Kleinjan et al.,^2004^; Zhang et al.,^2006^). The expression patterns governed by individual CREs join to make a complete picture. Importantly for the theory of morphological evolution, removal of single elements can alter the picture without destroying it. Therefore, mutations in CREs are an effective means for altering specific aspects of gene expression, for rewiring genetic regulatory networks, and for creating phenotypic diversity (Carroll,^2008^).

## PROVING A HYPOTHESIS

While there are certainly exceptions to the *cis*-regulatory hypothesis (Hoekstra and Coyne,^2007^), accumulating evidence supports that it is an important and often used mechanism of morphological change. Below are three examples from the literature that support the *cis*-regulatory hypothesis, and also introduce new hypotheses. These works operate under the notion that comparisons between closely related species are required to pinpoint the mutations that cause trait divergence.

### Trichome Loss in *Drosophila*

Stern and colleagues explore the genetic basis for the loss of the hair-like trichomes from the dorsal side of each segment in larval *Drosophila*. The naked cuticle phenotype independently evolved at least three times: in *D. sechellia*, a member of the *D. melanogaster* species group, and in two members of the *D. virilis* species group, which diverged from *D. melanogaster* approximately 60 million years ago. Through genetic mapping, they found that variation in the gene *shavenbaby* (*svb*) caused the naked cuticle trait in *D. sachellia* (Sucena et al.,^2003^). What's more, because *svb* expression correlates precisely with altered trichome patterns in divergent species, the authors reasoned that regulatory changes in *svb* expression account for the phenotypic differences. Indeed, a follow-up paper shows that changes in three separate *svb* enhancers, that individually make small changes in trichome patterning, cumulatively cause loss of dorsal trichomes (McGregor et al.,^2007^).

Why might *svb* be a site for convergent evolution? Stern describes *svb* as an “input–ouput” gene that lies at an integral position within its genetic regulatory network (Stern and Orgogozo,^2009^). If mutations were to occur in genes that regulate *svb* (i.e., *wingless*, *hox*, *hedgehog*), they would be more likely to cause pleiotropic effects. If mutations were to occur in any of the dozens of *svb*-regulated genes that comprise the trichome cell differentiation program, they may produce morphological changes that are too specialized or too minute to offer any selective advantage. On the other hand, changes within *svb* itself can specifically alter an entire module of differentiation genes. For these reasons, Stern argues that input–output genes are hotspots for evolutionarily relevant mutations.

### Pelvic Reduction in Sticklebacks

Here, Kingsley's group continues their search for mutations that cause an adaptive pelvic-reduced phenotype in the threespine stickleback fish, observed as a loss of the prominent serrated pelvic spine and hind fin skeleton (Shapiro etal.,^2004^; Chan et al.,^2010^). Fine genetic mapping localizes the cause of the phenotypic change to an intergenic region 23 kb upstream of *Pitx1*, a conserved “toolbox” transcription factor that, among other functions, regulates hindlimb and hindfin development in vertebrates. Within the intergenic region, the authors identify a 2.5 kb “*Pel*” CRE that drives pelvic-specific expression of GFP in transgenic fish, and rescues pelvic structures when fused with a *Pitx1* minigene in pelvic-reduced fish. Demonstrating the evolutionary importance of the region, 9 of 13 pelvic-reduced, and zero of 21 pelvic-complete natural stickleback populations have staggered deletions that overlap with the *Pel* CRE. They further show that the *Pitx1* locus resides within an unusually flexible region of the genome that may be susceptible to double-stranded DNA breaks and repair, offering a possible explanation for this case of convergent morphological convergent evolution. The authors postulate that fragile DNA may also contribute to parallel evolution of other phenotypes.

### Novel Spotted Wing Patterns in *Drosophila*

In this work, Carroll's group seeks the genetic mechanism for the evolution of a complex pattern of 16 vein-associated wing spots in *Drosophila guttifera*. They find that a single 277bp “*vein spot*” CRE upstream of *yellow*, a gene required for wing pigmentation, can drive GFP expression in the 16 spot pattern in transgenic *D. guttifera*. The absence of the *vein spot* CRE in *D. melanogaster* may be the reason that this species group does not bear wing spots. However, the *vein spot* CRE is present in *D. deflecta* which harbor a less complex variant of the *D. guttifera* pattern. Therefore, the cause of the different patterns in the two species groups may lie in the inducer of the *vein spot* CRE. In search of an inducer, the activity of the *vein spot* CRE was tested in the genetically well-defined *D. melanogaster*, and was found to direct reporter expression in a pattern similar to that of *wg*. Indeed, *wg* expression presages the spot patterns in *D. guttifera* and *D. deflecta*, and ectopic *wg* is able to drive ectopic black wing pigmentation in transgenic *D. guttifera*. Another important observation is that wing spots of multiple species always develop at physical wing landmarks: crossveins, campaniform sensillum, and vein fusion points.

From these and other data, the authors surmise that the *D. guttifera* wing spot pattern is a product of stepwise evolution. First, *wg* was expressed at a limited number of physical wing landmarks. Next *wg* became associated with pigmentation, partially through evolution of the *vein spot* CRE. Finally, *wg* evolved new sites of expression by adopting existing patterning information already present at additional physical wing landmarks. Of note is the fact that *wg* is a toolkit gene that has multiple functions in early development. *D. guttifera* can likely tolerate the extra spots of *wg* expression because they arise in the terminal stages of pupal wing development. Regardless, this view illustrates that an important feature of evolution is the layering of new information onto pre-existing gene activity patterns.

## A CONVERSATION WITH THE EXPERTS

**Fig. 2:**
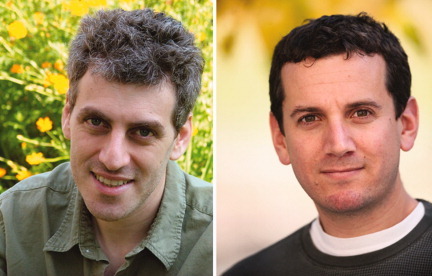
Left: David Stern, PhD, HHMI investigator and Professor of Ecology and Developmental Biology, Princeton University. Right: Michael Shapiro, PhD, Assistant Professor of Biology, University of Utah.

It remains to be seen whether the new hypotheses emanating from the works above will withstand the test of time. Moreover, as scientists discover additional principles of developmental biology, for example the guiding roles of epigenetics and noncoding RNAs, the theory of phenotypic evolution will continue to evolve. Evolutionary developmental biologists David Stern, PhD, and Michael Shapiro, PhD (Fig. 2), offer their perspectives on these and related topics in the field.

**Developmental Dynamics:** What is your lab's research focus?

**David Stern:** We have two foci at the moment. First, we study how development has evolved between closely-related *Drosophila* species to cause morphological divergence. Our most advanced project involves identifying the nucleotide changes of the *shavenbaby* locus that have led to divergence in larval morphology. Our finding, that multiple, small-effect mutations have accumulated in the *cis*-regulatory region of *shavenbaby*, has led us more recently to investigate questions of the structure and function of *cis*-regulatory regions. The second major focus of the lab involves studies of the evolution of behavior between closely-related *Drosophila* species. We are studying problems such as the evolution of courtship song. This is a new direction for the lab and these are early days. I am cautiously optimistic that we will have something interesting to say soon.

**Michael Shapiro:** We're interested in the developmental and genetic basis of evolutionary diversity among vertebrates. Our main focus is stickleback fish, which have served as important models for studies of behavioral, life history, and morphological variation for over a century. Beginning only approximately a decade ago, they have also emerged as important models for understanding the genetic and developmental changes that control ecologically relevant differences among populations. Sticklebacks are great models for this type of work because they show tremendous variation within and among species. Multiple species within the stickleback family have converged upon very similar adaptive phenotypes, giving us a unique chance to ask whether the same or different genes control similar evolutionary changes in independent evolutionary lineages.

**Dev Dyn:** What initially provoked your interest in this field?

**D.S.:** I came to evolutionary developmental biology through an unlikely path. I became interested in developmental biology while working on my PhD in the jungles of Malaysia on the behavior and evolution of soldier-producing aphids. Aphid colonies can produce multiple different phenotypic forms with the same genome and each individual aphid can develop along one of multiple possible developmental trajectories. Studying aphids in the field, I became increasingly interested in the mechanisms that allow aphids to develop into dramatically different body forms, such as winged versus unwinged forms, sexual versus asexual forms, and normal individuals versus soldiers. Also, the aphids I studied induced galls on trees. I spent most of my time in the field hunting down these galls. I think anyone who has worked on galls will tell you that it is easy to become obsessed with the question of how insects induce gall development on plants. This sterile field-dreaming about the mechanisms of development led me to the conclusion that I needed to learn more about the molecular mechanisms that generate diversity.

When I started looking for a postdoc in 1993, I had never studied developmental biology or molecular genetics and I was unaware of any of the recent literature on the evolution of development. I had read only work that approached development from an evolutionary perspective, such as the Maynard-Smith et al. review on constraints (Maynard Smith etal.,^1985^), Rupert Riedl's ideas on systems biology (Riedl,^1977^), and books such as *The Evolution of Individuality* (Buss,^1987^), and several of John Bonner's books (for example, Bonner,^1974^,^1988^). While these piqued my interest in development, they did not provide a useful guide to contemporary developmental biology.

I stumbled into Michael Akam's lab for a postdoc through a series of serendipitous encounters in Cambridge, UK, catalyzed by my friend and collaborator, William Foster, an expert on aphid biology. I wish I could say that I studied the literature closely and chose to work with Michael after careful consideration of the best workers in the field. But, the truth is, I was flying blind and I had a fantastic first discussion with Michael, which convinced me that I should work with him. Of course, I doubt that an informed search would have led me to a better choice of person to introduce me to the study of developmental biology with an evolutionary twist.

**M.S.:** My route to this field was also different than most others in evo-devo and evolutionary genetics—I started out in paleontology. As an undergraduate student at Berkeley, I took a few paleontology courses and one of my professors was kind enough to take me on a summer field trip to dig dinosaurs and their contemporaries in the Southwest, and I was instantly hooked. Fieldwork remains an important part of my lab's work to this day, and I think it's important for student and postdocs to see their organism of choice in the real world rather than just in a tube in the lab. I gradually became more interested in the developmental processes that led to the types of diversity I saw in the fossil record, and for my dissertation project, I studied the developmental basis of limb reduction in a genus of Australian skinks that had different species with 5, 4, 3, or 2 fingers and toes. These lizards provided a great opportunity to find some molecular correlates of digit loss—for example, changes in the expression pattern of a key limb development gene—but we couldn't really get at the genetic basis of these limb changes because we couldn't interbreed the different species to take a forward genetic approach. In other words, we could see that gene expression was changing, but we couldn't definitively tell whether there were changes to the candidate genes we had chosen, or changes to an upstream signal affecting our candidates.

As I was getting close to finishing my dissertation, one of my committee members told me about a great new project that David Kingsley's lab had recently started on stickleback genetics, and that some of the populations of these fish were missing their “limbs.” The big advantage of sticklebacks is that you can do genetics and let the fish tell you which genes are important, rather than using a candidate approach like we did with the lizards. From there, we can test the developmental roles of the genes we identify. To me, this was an incredibly powerful approach to understanding which genes are actually important in adaptive evolution.

**Dev Dyn:** What three papers have most impacted your research and why?

**D.S.:** Limiting this response to anything close to three papers is difficult because I have learned so much from a huge number of papers. So, I will focus on three papers that are not usually on the radar of developmental biologists, but which influenced my thinking significantly.

As an undergraduate, I was fascinated by circadian rhythms and by *Drosophila* courtship song and I have a very clear memory of reading Zehring et al. (1984) when I was working in Chip Aquadro's lab at Cornell University in the 1980s. During my undergraduate years, I was fascinated by both genetics and the evolution of behavior and I very much hoped to combine these interests. This paper, which demonstrated that the aberrant behavior of a *period* mutant could be rescued by P-element transformation with the native locus, provided a clear indication of how to go about this kind of work. While this work has provided inspiration, even since my earliest days in science, it took me many years to find a tractable way to address these questions in an evolutionary framework. We are now undertaking this work on the evolutionary genetics of behavior and I cannot begin to express the thrill of finally working on questions that I have carried with me since my undergraduate days.

I could have chosen several of Cathy Laurie's extraordinary papers as one of my top three. One paper stands out (Stam and Laurie,^1996^), because it was the first paper that provided a clean experimental dissection of a naturally occurring evolutionary variant. Previous work had implicated primarily an amino-acid polymorphism as the major, if not the only, determinant of variation in alcohol dehydrogenase activity in natural populations of *D. melanogaster*. Through a series of elegant molecular dissection experiments, this paper clearly showed that multiple additional polymorphisms, including multiple noncoding polymorphisms, within the *Adh* gene contribute to levels of *Adh* expression. I think this work was far ahead of its time and demonstrated the clear importance of *cis*-regulatory variation. Unfortunately, I think this work has been somewhat overlooked by workers in the field of evolutionary developmental biology.

I first read Boucher etal. (1992) when I started work on my book, approximately 5 years ago. I was shocked by the predictable spread of mutations in a population of HIV during antiviral treatment of infected individuals. While this extraordinary level of predictability probably results, at least in part, from the relatively small and simple genome of HIV, these observations inspired me to be open to the possibility that such predictability might be widespread. These, and similar, observations led me to think about why this predictability might exist. This is a problem I am still pursuing and which I think will be one of the unifying themes of evolutionary developmental biology in the future.

**M.S.:** The first paper we read in my undergraduate evolutionary biology class was “Evolution and Tinkering” by François Jacob (1977). The message was quite simple, but it had a profound impact on the way I thought about biology. In short, Jacob's argument was that evolution “acts” more like a tinkerer than an engineer, selecting for modifications to existing genes and processes to generate variation rather than designing new phenotypes from scratch. The paper was published in^1977^, long before the genomics era, yet it's still highly relevant. We now know, for example, that a rather large set of critical developmental genes—the so-called “genetic toolkit”—is highly conserved throughout the Metazoa. A lot of differences among organisms have more to do with when and where these genes are expressed, and slight tweaks to the coding regions, rather than complete overhauls of gene function or the evolution of completely new gene families. What we consider the evolution of novel structures often involves the redeployment of existing gene networks in new ways, not the evolution of entirely new genes. Jacob's paper is now the basis for the first writing assignment in the undergraduate course that I teach.

As an undergraduate and graduate student, I became very interested in vertebrate limb development and the developmental basis of limb diversity, and I was excited by the work of Pere Alberch and Neil Shubin (Shubin and Alberch,^1986^), and John Saunders (Saunders,^1948^). I was really blown away by the work coming out of Cliff Tabin's lab (Riddle et al.,^1993^), and a few other labs, that connected molecular mechanisms to the embryological processes that others had observed. This work gave me hope that we could eventually identify the genetic targets of evolutionary “tinkering” that led to diversity in vertebrate limbs and the skeleton in general.

**Dev Dyn:** Work from David's lab supports the theory that genes at integrative positions in developmental networks are genetic “hotspots” for phenotypic evolutionary changes. David, do hotspots reflect neutral processes or natural selection? Mike, what is your perspective on this theory?

**D.S.:** Since all neutral mutations fix in populations at the same rate, the only way hotspots could reflect neutral processes is if evolutionary hotspots were mutational hotspots. Since I don't think this is a good general explanation, this leaves only natural selection. There must be something about the mutations that occur at particular genes in genetic networks that make them more favorable, on average, than mutations at other genes in the network.

I think there are at least two facts that lead to this bias. The first is that mutations at different genes in a network can have widely different pleiotropic effects. In our work on *shavenbaby*, most, or perhaps all, mutations in genes that act upstream of *shavenbaby*—and that would result in similar phenotypic changes—would be very likely to have pleiotropic effects on segmentation generally. The second fact is that some genes “control” entire modules of morphogenesis. For example, *shavenbaby* activity regulates a large number of downstream effector genes that build trichomes. No individual gene acting downstream of *shavenbaby* can induce this entire module of morphogenesis.

*Shavenbaby* may be an extreme case, but the regulatory network containing *shavenbaby* is certainly similar, in overall architecture, to how we tend to envision hierarchies of gene regulation underlying development. That is, I think hotspot genes are probably abundant and that hotspots arise from the architecture of the genetic network. Natural selection is very discriminating and, if I were to go out on a limb, I think selection has led to a superabundance of mutations causing phenotypic evolution at hotspots.

**M.S.:** I think the idea of “hotspots” in integrative network positions is an important and testable hypothesis. David's work on *svb* certainly supports the idea, and to a lesser extent, the *Pitx1* story in sticklebacks is consistent with it as well. I agree with David that we might see an overrepresentation of changes at these integrative positions, but I'd also like to see some additional examples before deciding that this is a predominant and predictable mechanism of evolutionary change. Examples from the evolution of certain phenotypes, such as changes in the sequence and expression of genes that control evolutionary variation in vertebrate pigmentation, provide potential counterexamples to part of this hypothesis. For instance, *Mc1r* is unquestionably a hotspot of repeated mutation leading to evolutionary variation (Hoekstra,^2006^), but I would argue that it does not hold the same kind of integrative network position that David's work on *svb* demonstrates.

***Dev Dyn:** David Kingsley's lab showed that independent mutations in pitx1 contribute to pelvic variation in natural populations of threespine stickleback fish. Interestingly, the mutations occur in a genome region that is thought to be more susceptible to deletions (Chan etal.*,^2010^). *Might this reflect a general trend?*

**D.S.:** In general, natural selection, rather than mutation, is expected to be a much stronger force influencing the frequency of alleles in populations. So, the specific question about fragile regions is whether they are more likely to throw up adaptive variation than are other kinds of mutations. This seems rather unlikely to me. The most compelling data from studies of species difference in *Drosophila*, including some of our unpublished work, indicates that normally phenotypic evolution is not driven by unusual kinds of mutations.

It is unlikely that large populations are often mutation limited; they usually contain an abundance of mutations available for natural selection to act upon. However, species with smaller population sizes, like Darwin's finches and, perhaps, stickleback populations that invade new lakes, may be mutation limited. In this case, the increased frequency of mutations in fragile regions may bias evolution toward use of these mutations. Also, if selection acts strongly, like it appears to do in these stickleback populations, then mutations of rather large effect—such as mutations that cause dramatic changes to gene function—may tend to be selected more often than are mutations of more subtle effect.

I think that the specific ways in which genomes have evolved, and thus the resulting assemblage of mechanisms that guide development, is dependent on quirks of population history. This is a major theme of my recent book *Evolution, Development, & the Predictable Genome* (Stern,^2010^).

**M.S.:** Again, I think we know relatively little about the nucleotide-level changes that are responsible for evolutionary diversity in metazoans. This is especially true for regulatory (as opposed to coding) changes, which likely account for a great deal of phenotypic diversity. With this in mind, I don't think we can yet determine whether the types of mutations seen in the *Pitx1* regulatory region are common or unusual. These types of fragile regions are almost certainly present across metazoan genomes, and it will be interesting to see if the distribution of these sites corresponds to the genes that underlie adaptive variation. While fragile sites are probably subject to higher mutation rates, what's not clear is whether a disproportionate number of these mutations are selected and persist. It's also worth noting that transposable elements can potentially have similar effects on the genome as fragile sites. In addition to disrupting genes or their regulatory regions, these elements can lead to deletions in the genome, as well as unequal recombination. Transposable elements are likely important factors in human disease, and in the stunning diversity in the domestic dog, whose genome is littered with a particularly active type of these elements. It seems likely to me that these elements could play a role in natural diversity as well.

***Dev Dyn:** microRNAs are a means of manipulating gene expression that are unique to metazoans. As such, it has been put forth that they have been important for the evolution of complex body plans (Christodoulou et al*.,^2010^). *What are your thoughts about miRNAs, or other noncoding RNAs, in evolution?*

**D.S.:** My thoughts on this topic are similar to my thoughts on the contributions of all kinds of genomic changes to phenotypic evolution. Namely, we currently have too limited a set of data collected in a sufficiently unbiased manner to answer any of these questions with rigor. At the moment, we have examples of miRNA targets that have evolved in domesticated populations, but to my knowledge, no examples of miRNAs themselves, or miRNA targets in natural populations, that have contributed to natural variation. Given the fact that miRNAs obviously have evolved, it is almost certainly just a matter of time before such examples are found. I think it is probably unlikely that evolution of miRNAs played a large role in phenotypic evolution in natural populations, or we would probably have already discovered some examples.

But, I must emphasize that we are dealing currently with a small sample of the mutations known to contribute to phenotypic evolution in natural populations, and, for various reasons that I have discussed in other forums (Stern and Orgogozo,^2008^), there are good reasons to believe that the vast majority of our current examples come from biased studies. For example, if an investigator maps a phenotypic difference to a genomic region carrying an obvious protein-coding candidate gene, then are they likely to look for alternative explanations? At the moment, the most compelling evidence for the molecular changes contributing to phenotypic evolution in the wild and between species does not include examples of miRNAs or other noncoding RNAs. But, I expect that they will be found eventually.

**M.S.:** I agree with David's caution and the biases that he highlights. Most of the work in evolutionary molecular genetics tends to focus on genes, either their coding regions or regulatory elements. Little is known about the evolutionary roles of noncoding RNAs, not because we think they aren't important, but rather because the field has paid little attention to the topic thus far.

**Dev Dyn:** How does the field of evolution shed light on mechanisms of development?

**M.S.:** As a colleague once told me, nature has been doing mutagenesis on a scale that even NIH funding can't touch. The enormous organismal variation we see in the wild must have resulted, at some point, from changes in developmental programs, and this giant, worldwide mutant screen gives us a tremendous resource with which to explore the molecular basis of developmental processes. Evolutionary studies can shed light on the generality of developmental processes—are the same genes always involved in the generation of similar phenotypes across different groups?—and can illuminate more basic developmental mechanisms as well. For example, the threespine stickleback was an ideal organism for finding the *cis*-regulatory region controlling pelvic expression of *Pitx1* because this species shows dramatic, natural variation in pelvic morphology. This evolutionary study resulted in the identification of a *cis*-regulatory element that is surely present, yet has not been identified, in the more traditional models of vertebrate development.

On a more practical note, over the past few decades, comparative studies of developmental genetics have also led to the realization that the same gene networks are present throughout metazoans. This remarkable conservation has led to the adoption of a wide range of organisms, not just mice, as genetic models for normal and abnormal human development and variation.

**D.S.:** Perhaps the most important way that evolution sheds light on mechanisms of development is to recognize that all genomes have evolved, both in response to natural selection and through neutral processes. Genome architecture and function must reflect this history of selection and drift. I sometimes feel that there is a subtle tendency in the field of developmental biology to view the genome as an engineered product, instead of viewing the genome as the product of evolution, a product of bricolage, as François Jacob put it. Viewing the genome as a product of a long history of natural selection and drift immediately flags three important things that we should look for in the genome.

First, the contingency of evolution suggests that “solutions” to development that we observe today may appear like odd ways of doing things. Development may take three rights to make a left. For example, the *Drosophila melanogaster* male genitalia undergoes a 360° clockwise rotation during development to end up precisely where it would have ended up without rotating. Presumably this reflects an evolutionary history during which an ancestor of *D. melanogaster* developed with a genitalia that rotated 180° to facilitate back-to-back or face-to-face mating. There is, obviously, a set of developmental mechanisms in place to accomplish the 360° rotation of the genitalia that is, currently, a complete waste. But evolution built upon what was already there and now the poor fly is stuck with these wasteful mechanisms. It is difficult to argue that this is the optimal way for a genitalia to develop.

Second, much of the genome of most species has evolved through neutral processes. In recent years, Michael Lynch has been asking how, precisely, neutral processes might influence genome complexity, such as the origin of genes, the prevalence of gene duplication, and the like. His general result is that neutral processes provide an abundant source of genome complexity. These are tricky hypotheses to test robustly, but, again, the major point is that many aspects of the structure and function of genomes may not reflect adaptation according to any measure of optimality.

Third, against the backdrop of historical contingency and genetic drift, natural selection plays a widespread and discriminating role in sculpting genome structure and function. The extent of natural selection on genome function has only recently started coming into clear focus. For many years, most molecular evolutionary biologists assumed that selection (both negative and positive) acted primarily on nonsynonymous amino acid substitutions and that most of the noncoding regions of genomes were subject to neutral or near-neutral processes. It is now clear that natural selection (both negative and positive) is pervasive throughout many noncoding regions of metazoan genomes (Andolfatto,^2005^). Many genomic regions show strong patterns of evolutionary conservation that cannot be explained by current functional assays. For the genes I have worked on, on a good day I like to think that we can explain maybe 5–10% of the evolutionarily conserved DNA associated with these genes.

**Dev Dyn:** What are some up-and-coming techniques that promise to propel the evo-devo field forward?

**M.S.:** My perception is that evolutionary developmental biology and evo-devo increasingly mean evolutionary genetics, and this is especially true for those of us interested in microevolutionary questions. With this in mind, I think the rapidly evolving set of genomics techniques are starting to have a huge impact on the field, and this impact will only increase. Genomics is hardly a new set of techniques, of course, but until recently these tools were largely unavailable to those of us studying weird organisms. Due to funding constraints, labs studying the genetic basis of human disease were the main beneficiaries of techniques that examined variation across the genome, but we're now starting to see studies emerge involving the genomic basis of adaptation in humans and a modest number of other organisms. The precipitous decrease in sequencing costs is making it easier (but still by no means trivial) to sequence, assemble, and annotate a reference genome for your favorite organism. This reference, coupled with comparative data from other individuals, populations, or species can be an important entry point to finding genomic regions under selection or associated with important phenotypes.

**D.S.:** Overall, we should look for dramatic improvements in the ability to manipulate gene structure and function in nonmodel systems. Improvements in transgenic technology are opening up opportunities to manipulate gene function in systems such as crustacea and a diversity of insects. I think we should keep an eye on engineered zinc fingers and TAL effectors for the ability to target manipulations to almost any genomic region in a wide variety of systems. These systems are gaining ground in model systems, and it is only a matter of time before they are transferred to nonmodel systems. These tools open up the possibility of performing homologous recombination in a diversity of organisms, which, really, will be the gold standard for how to test evolutionary hypothesis of functional molecular evolution.

I agree with Michael that genomics technologies are vital to progress on nonmodel systems, and I will reiterate his point that assembling and annotating new genomes is far from trivial, even with a big pot of money. In my opinion, the scale of this problem has been grossly underestimated; high sequence throughput does not equal easy genome assembly.

Finally, one area where high-throughput sequencing is making a big difference fast is in genetic mapping experiments. I think next generation sequencing, combined with some new approaches to thinking about genetic mapping, will quickly make most other genotyping platforms obsolete and will provide high resolution genetic mapping in a wide and interesting diversity of systems.

**Dev Dyn:** What are some exciting ideas that are emerging?

**M.S.:** I'm intrigued by the potential roles of epigenetics in the evolution of development. Strong evidence is emerging that nongenetic changes such as DNA methylation, which can have profound effects on gene expression and transposon activity, can be inherited transgenerationally. Epigenetic marking can occur in response to environmental stimuli, so environmental factors can potentially influence development for multiple generations. If these epigenetic changes occur in response to new habitats, then epigenetic process could play a key role in generating new phenotypes during adaptive radiations. These ideas are not new, but we still have few empirical examples of how these processes might work.

**D.S.:** In addition to questions that I have discussed above, such as our ignorance of the functions of large genome regions, I will add a few more favorites. First, the history of evolution is primarily a history of changes in the shapes and sizes of organs. We currently have almost no understanding of the mechanisms underlying evolutionary changes in shape and size. This ignorance reflects partly our still elementary understanding of shape and size control in model systems. I think that serious progress on these problems will require first some dedicated work on model systems.

Second, there has been vanishingly little work done on the role of precise changes in gene expression levels and timing to evolutionary transitions. Most work has focused on rather dramatic changes in gene expression patterns. This emphasis reflects, in part, what has been possible. As methods for quantifying temporal patterns of gene expression improve, I expect that we will see more work exploring the role of subtle changes in the temporal dynamics of gene expression in evolutionary transitions.

Third, based on some of our recent observations (Frankel et al.,^2010^), I expect that metazoan genomes encode lots of apparently redundant enhancers. Our recent findings suggest that some of the apparently redundant enhancers have been evolutionary conserved and contribute to phenotypic robustness. The developmental sources of robustness—or canalization, as Waddington called it—have been mysterious for decades and I think we are just beginning to get a window into these mechanisms. Robustness seems to reflect selection acting in the real world, a world of variable temperature, variable sources of nutrition, and of variable genomes. Studying development in the laboratory, under tightly controlled conditions, means that we are studying only a tiny fraction of how the genome functions. I foresee an explosion of studies into how the genome responds to variable environments and whether and how this contributes to developmental evolution.
